# Factors affecting femoral rotational angle based on the posterior condylar axis in gap-based navigation-assisted total knee arthroplasty for valgus knee

**DOI:** 10.1371/journal.pone.0197335

**Published:** 2018-05-15

**Authors:** Sung-Sahn Lee, Yong-In Lee, Dong-Uk Kim, Dae-Hee Lee, Young-Wan Moon

**Affiliations:** Department of Orthopedic Surgery, Samsung Medical Center, Sungkyunkwan University School of Medicine, Seoul, South Korea; Universidad de Zaragoza, SPAIN

## Abstract

**Background:**

Achieving proper rotational alignment of the femoral component in total knee arthroplasty (TKA) for valgus knee is challenging because of lateral condylar hypoplasia and lateral cartilage erosion. Gap-based navigation-assisted TKA enables surgeons to determine the angle of femoral component rotation (FCR) based on the posterior condylar axis. This study evaluated the possible factors that affect the rotational alignment of the femoral component based on the posterior condylar axis.

**Materials and methods:**

Between 2008 and 2016, 28 knees were enrolled. The dependent variable for this study was FCR based on the posterior condylar axis, which was obtained from the navigation system archives. Multiple regression analysis was conducted to identify factors that might predict FCR, including body mass index (BMI), Kellgren-Lawrence grade (K-L grade), lateral distal femoral angles obtained from the navigation system and radiographs (NaviLDFA, XrayLDFA), hip-knee-ankle (HKA) axis, lateral gap under varus stress (LGVS), medial gap under valgus stress (MGVS), and side-to-side difference (STSD, MGVS − LGVS).

**Results:**

The mean FCR was 6.1° ± 2.0°. Of all the potentially predictive factors evaluated in this study, only NaviLDFA (*β* = −0.668) and XrayLDFA (*β* = −0.714) predicted significantly FCR.

**Conclusions:**

The LDFAs, as determined using radiographs and the navigation system, were both predictive of the rotational alignment of the femoral component based on the posterior condylar axis in gap-based TKA for valgus knee. A 1° increment with NaviLDFA led to a 0.668° decrement in FCR, and a 1° increment with XrayLDFA led to a 0.714° decrement. This suggests that symmetrical lateral condylar hypoplasia of the posterior and distal side occurs in lateral compartment end-stage osteoarthritis with valgus deformity.

## Introduction

Valgus knee deformity has several challenges, including lateral condylar hypoplasia, lateral cartilage erosion, and tightening of the lateral structures (lateral collateral ligament, posterolateral capsule, popliteus tendon, and iliotibial band). For these reasons, total knee arthroplasty (TKA) for valgus knee deformity is challenging [1, 2].

**Fig 1:**
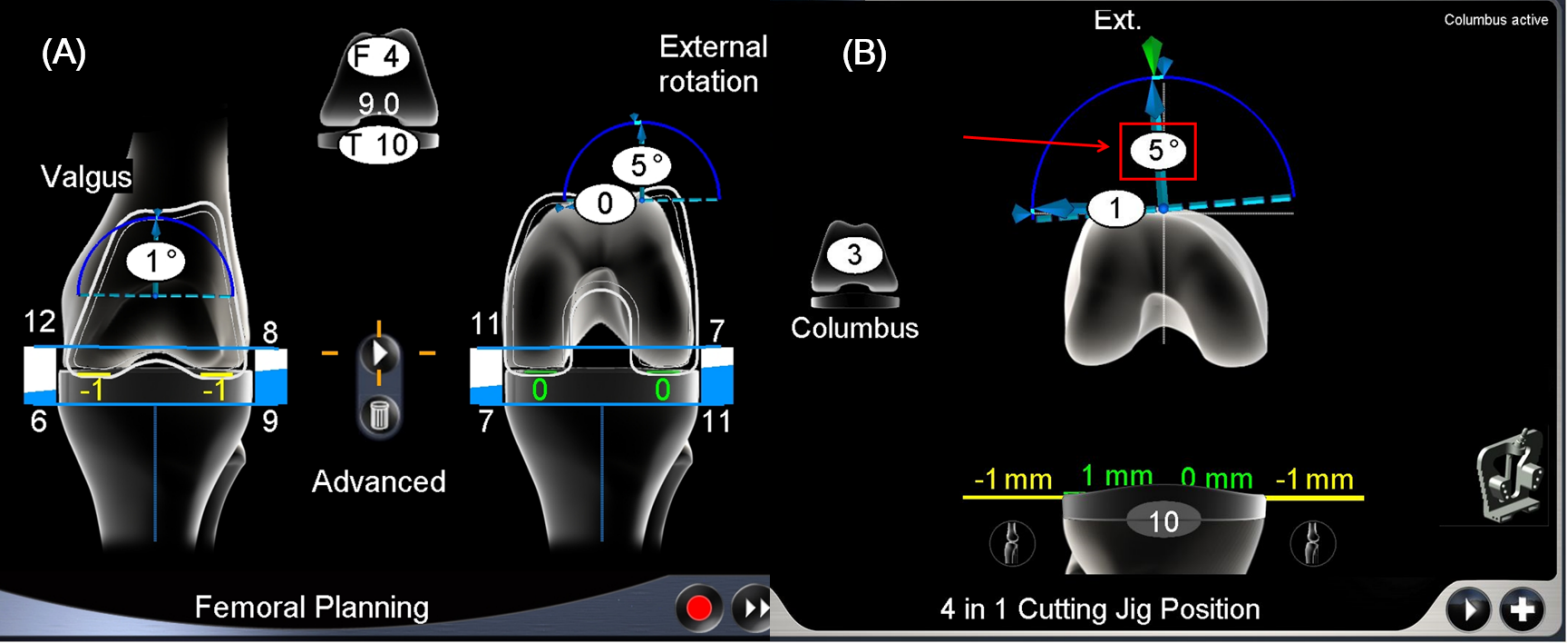
Adjustment of femoral component rotational alignment. (A) To obtain a rectangular gap in the navigation femoral planning step, the femoral component rotation based on the posterior condylar axis and the varus-valgus angle were adjusted. A difference of <2 mm between the lateral extension/flexion gap and the medial extension/flexion gap was considered acceptable. (B) After distal femoral resection, the AP femoral cutting jig is located using the determined value during the planning step. The rotational position of the AP femoral cutting jig is displayed in real time. The *arrow line* indicates the actual femoral component rotation.

In gap-technique TKA, the tibia is resected in advance, and anterior and posterior cuts of the femur are performed parallel to the tibial cut. As a consequence of this procedure, the rotation of the femoral component can vary freely with the restriction of the soft tissue release [3, 4]. Use of a gap technique-based navigation system allows surgeons to quantify femoral component rotation based on the posterior condylar axis (Fig 1) [4, 5].

**Fig 2:**
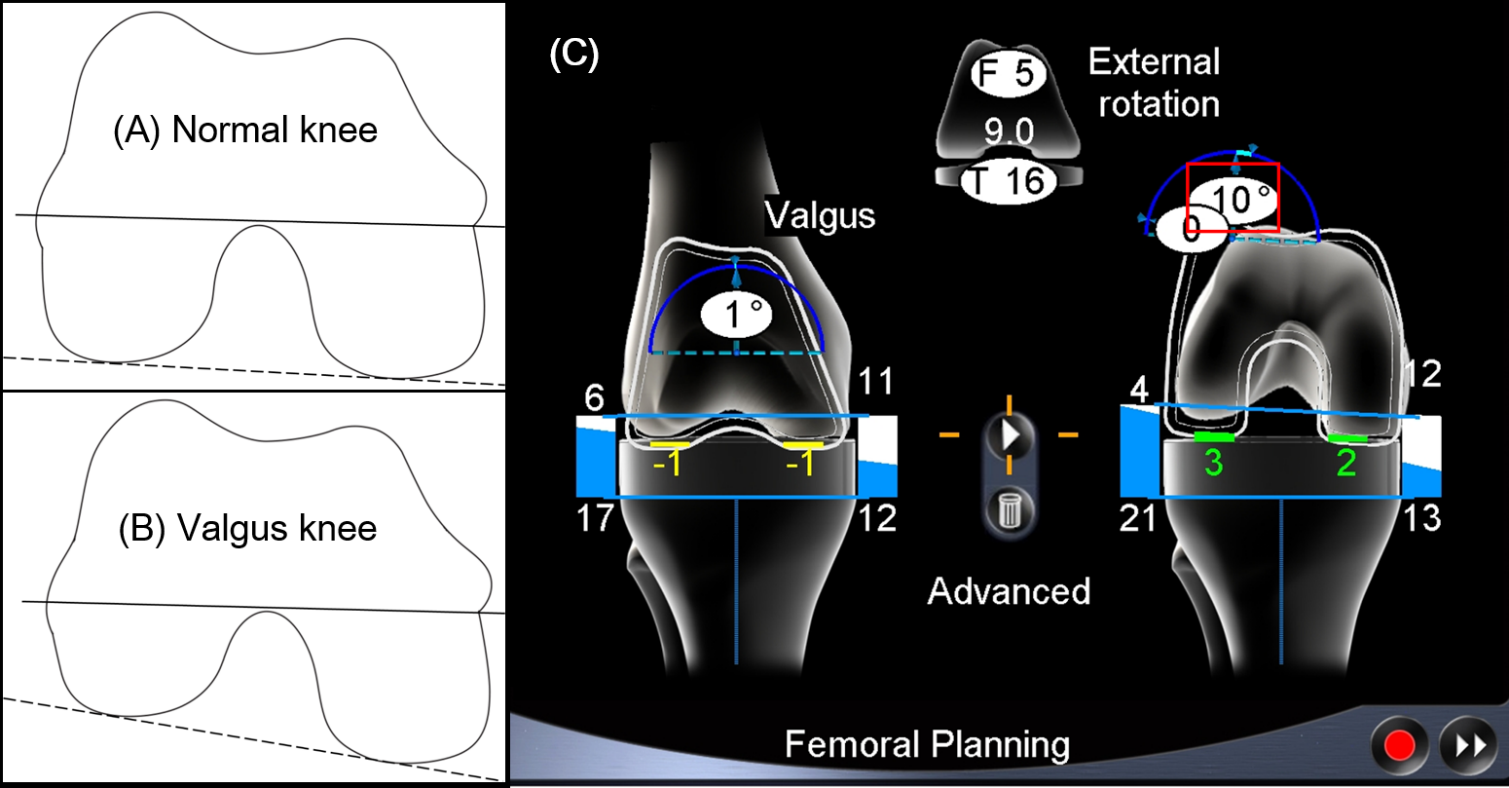
Excessive external rotation is needed for valgus knee TKA. (A) The surgical transepicondylar axis (*solid line*) connects the medial sulcus to the lateral epicondyle. The posterior condylar axis (*dashed line*) is the tangent of the posterior part of the medial and lateral condyle. (B) Posterior lateral condylar hypoplasia induces a more internal rotation of the posterior condylar axis relative to the surgical transepicondylar axis. (C) In this case, 10° external rotation of the femoral component from the posterior condylar axis is needed to achieve a rectangular flexion gap.

As the posterior lateral condylar hypoplasia of the femur induces internal rotation of the posterior condylar axis, excessive external rotation of the femoral component can occur when performing TKA for a valgus knee deformity with a navigation system; this can cause surgeons to doubt whether the measurement is correct (Fig 2). Thus, the purpose of this study was to identify factors that significantly affected the femoral component rotation in valgus deformity by using a navigation system. We hypothesized that the severity of the lateral condylar bony tissue deformity is the primary factor that affects femoral component rotation.

## Materials and methods

### Study design and subjects

This study is a retrospective database-based cohort study. We evaluated patients who underwent primary TKA between January 2008 and May 2016. The inclusion criterion was patients who underwent TKA using a navigation system for end-stage lateral compartment osteoarthritis with valgus deformity. The exclusion criteria were as follows: 1) patients with a history of prior knee surgery or a diagnosis of posttraumatic osteoarthritis, 2) patients with unavailable radiographs and navigation data, and 3) patients who underwent lateral soft tissue release (iliotibial band, popliteus muscle, and lateral collateral ligament) after review of operative records and intraoperative navigation data to evaluate the effect of soft tissue deformity. For this study, 28 knees in 26 patients were included in this study (9 knees in 9 men and 19 knees in 17 women). The mean (range) patient age and body mass index (BMI) were 66.7 ± 5.3 years (55–76 years) and 25.1 ± 2.7 kg/m^2^ (19.8–31 kg/m^2^), respectively.

The protocol used to evaluate radiographic findings and intraoperative navigation data was approved by the investigational review board of Samsung Medical Center of South Korea (SMC2017-05-049). All the patients provided written informed consent.

### Procedures

**Fig 3:**
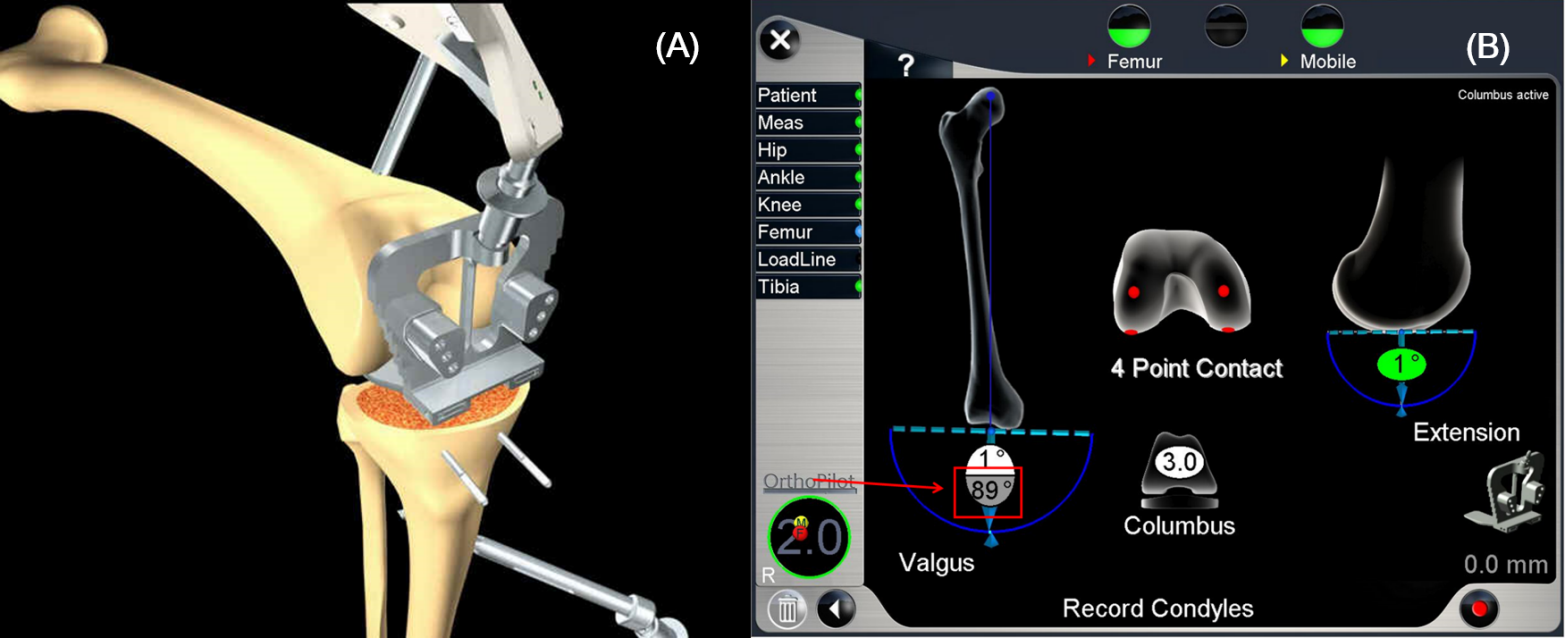
Obtaining NaviLDFA. (A) The angle between the true mechanical axis of the femur obtained using hip and knee kinematic analysis and distal femoral joint surfaces from the exact 4-point contact with a check plate. (B) The navigation system displays the quantified angle. This angle (*arrow line*) represents the lateral distal femoral angle from the navigation system (NaviLDFA).

All the surgeries in this study were performed by the senior author by using an image-free computerized navigation system (OrthoPilot; B. Braun, Aesculap, Tuttlingen, Germany). The patients received either an Ultra-Congruency type of E-motion or a Columbus prosthesis (B. Braun, Aesculap, Tuttligen, Germany). The arthrotomy was made using a medial parapatellar approach. To identify hip, knee, and ankle joint centers, the kinematic and the required anatomically selected points were registered. Upon completion of the registration process, the femoral alignment was calculated using a 4-point contact with a check plate on the distal femur. The navigation program calculated the angle between the true mechanical axis of the femur by using the pre-registered data (intersecting line from the hip center to the knee center) and the distal femoral joint surface, which reflected both bone and cartilage status; this angle represented the lateral distal femoral angle obtained from the navigation archives (NaviLDFA; Fig 3). Proximal tibial cutting was performed in a plane perpendicular to the mechanical axis of the tibia. A slide ruler with a laminar spreader (maximum right-hand grip, 40 kg) was used to identify the medial and lateral gaps at 90° flexion and extension. Femoral planning included component size, rotation, and the amount of posterior bone cutting required for a balanced gap. Measurement of the external rotation was considered the FCR (Fig 1). Gap balancing was considered acceptable when the difference between the lateral extension/flexion gap and the medial extension/flexion gap was <2 mm. Femoral bone cutting and implantation of the prosthesis were performed. Patella resurfacing was not performed.

### Measures

**Fig 4:**
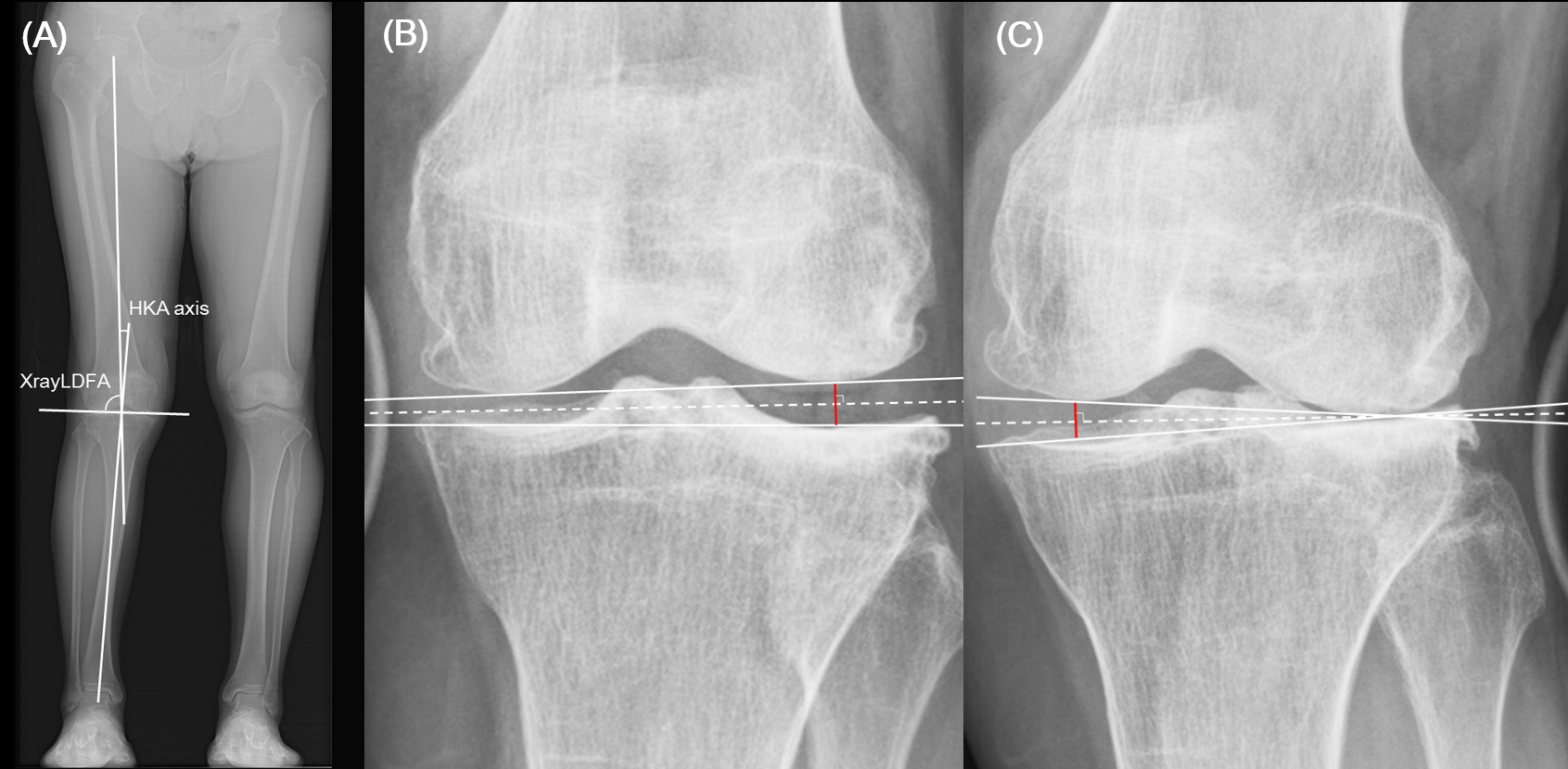
Measurement of HKA Axis, XrayLDFA, LGVS, and MGVS. The HKA axis and XrayLDFA were measured using a preoperative whole-leg standing radiograph (A). The HKA axis was measured using intersecting lines from the femoral and tibial mechanical axes. The XrayLDFA was a superolateral angle measured by intersecting the femoral mechanical axis line and distal femoral joint line. LGVS and MGVS were measured from varus (B) and valgus (C) stress radiographs, applying 130 N. The *dashed line* is the bisector of the angle between the distal femoral and proximal tibial joint lines (*black solid lines*). LGVS was measured from the lowest point of the lateral femoral condyle to its corresponding point on the tibial joint line (*red solid line*, drawn perpendicular to the *dashed line*). MGVS was measured using a similar method.

Potential predictive factors of FCR were evaluated in this study, including BMI, bony tissue deformity, and soft tissue deformity. Factors used to evaluate bony tissue deformity were the Kellgren-Lawrence grade (K-L grade) [6] of the lateral compartment, NaviLDFA, the LDFA obtained from radiographs (XrayLDFA), and the hip-knee-ankle axis (HKA axis). The XrayLDFA and HKA axis were both measured using preoperative whole-leg standing radiographs. A positive value for the HKA axis indicated valgus deformity. Soft tissue deformity was quantified using preoperative varus and valgus stress radiographs of the knee joint in full extension with the assistance of a Telos device (Telos, Griesheim, Germany; 130-N load), measurements of soft tissue deformity were consisted with the lateral gap under varus stress (LGVS), the medial gap under valgus stress (MGVS), and side-to-side difference (STSD, MGVS − LGVS; Fig 4).

**Fig 5:**
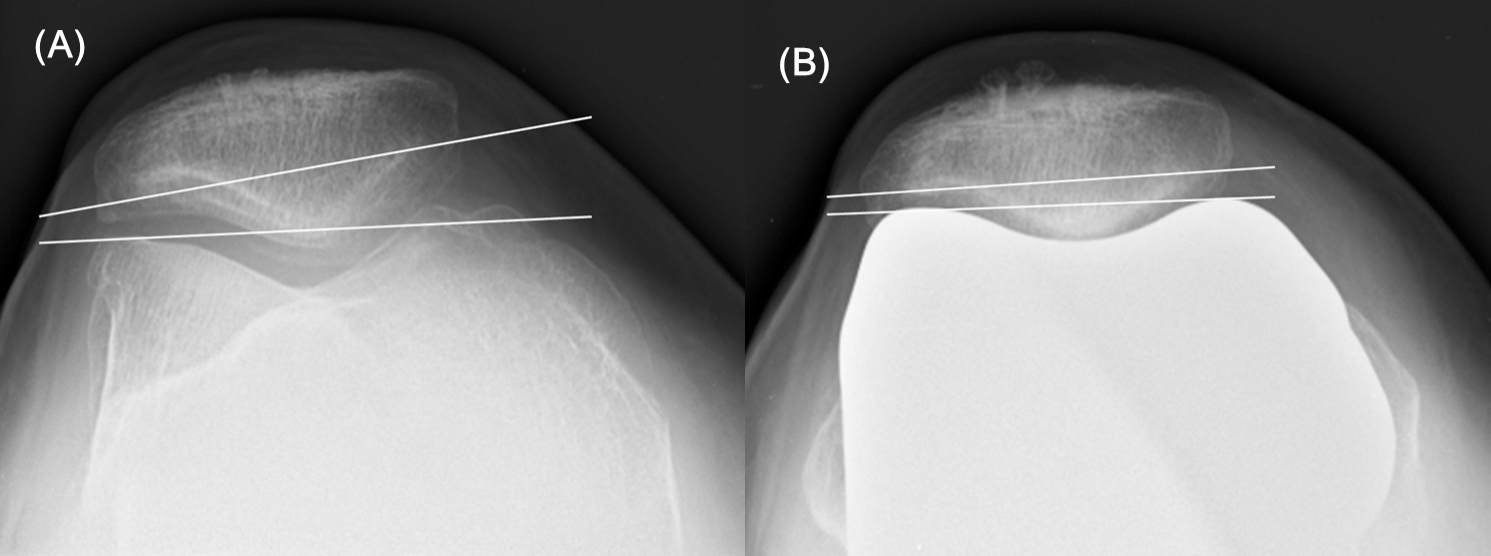
Patellar tilt angle. (A) The preoperative patellar tilt angle is defined as the angle between the equatorial line of the patella and the line connecting the anterior limits of the femoral condyles in the Merchant view. (B) The postoperative patellar tilt angle is measured using the same method but with a line connecting the anterior limits of the femoral component instead of the femoral condyles.

Both LDFAs and the HKA axis were compared to analyze the correlation between femoral coronal obliquity and the degree of valgus deformity. Preoperative and postoperative patellar tilt angles (Fig 5) were measured in the Merchant view and used to evaluate the malposition of the femoral component rotation. A positive value indicated opening toward the medial side of the patella. Patellar tilt angles of >10° were considered outliers [7]. The postoperative HKA axis was also measured on whole-leg radiography to evaluate the improvement of the coronal plane axis. Postoperative measurements were obtained from radiographs taken at 1-year follow-up. The variables described earlier were all measured to the nearest 0.1° by using a PACS system (Centricity; General Electric, Chicago, Illinois). Radiographs were evaluated by two independent orthopedic surgeons to verify interobserver reliability. Intraobserver reliability was checked by having the observers repeat the same measurements 1 month later. The intraclass correlation coefficient (ICC; two-way mixed effects model, consistency definition) was used to quantify both interobserver and intraobserver reliability. All interobserver and intraobserver ICCs showed excellent agreement regarding radiographic measurement reliability (>0.80).

### Statistical analysis

The Shapiro-Wilk test was used to check the normality of distributions. Means and standard deviations were calculated for all the variables. Paired *t* tests were used to evaluate differences between both LDFAs, the preoperative and postoperative HKA axes, and the patellar tilt angle. Pearson correlation analysis was used to identify correlations between both LDFAs and the preoperative HKA axis. Backward multiple regression analysis was used to evaluate the following independent variables associated with FCR: BMI, NaviLDFA, XrayLDFA, preoperative HKA axis, LGVS, MGVS, STSD, and K-L grade. Due to multicollinearity, multiple regression analysis was performed twice with each LDFA, rather than both NaviLDFA and XrayLDFA, as independent variable. A stepping method criterion with a probability of F to remove ≥0.10 was used. *P* values of <0.05 were considered statistically significant. All data were analyzed using the Statistical Package for the Social Science software version 20.0 (SPSS, Inc., Chicago, IL, USA).

## Results

The mean and standard deviation values were 84° ± 2° (range, 79–88°) for NaviLDFA and 85.2° ± 1.6° (range, 82.1–88.8°) for XrayLDFA. NaviLDFA was significantly less than XrayLDFA (*P* < 0.001), and a significant correlation was found between the two variables (*r* = 0.783, *P* < 0.001). Both LDFAs were correlated with the preoperative HKA axis. (*P* value of NaviLDFA = 0.005, XrayLDFA = 0.044) Table 1 represented correlation coefficients between FCR and all potential predictors. The mean and standard deviation of the dependent and independent variables are described in Table 2. The first multiple regression analysis revealed that the NaviLDFA significantly predicted with FCR (*r*^2^ = 0.447, *P* < 0.001), the second analysis with XrayLDFA showed a similar result (*r*^2^ = 0.324, *P* = 0.002). NaviLDFA showed better prediction of the FCR variation than XrayLDFA (44% vs 33%). None of the other factors tested was a significant predictor of FCR (Table 3 and Table 4).

**Table 1:** Correlations between FCR and all potential predictors.

	FCR	BMI	Preoperative HKA axis	K-L grade	Navi LDFA	Xray LDFA	LGVS	MGVS
BMI	-0.173							
Preoperative HKA axis	0.349	-0.180						
K-L grade	0.089	0.185	0.056					
**NaviLDFA**	**-0.668**^**a**^	0.272	**-0.520**^**b**^	-0.156				
**XrayLDFA**	**-0.569**^**c**^	0.248	**-0.383**^**d**^	-0.194	**0.783**^**e**^			
LGVS	-0.004	-0.023	-0.019	-0.247	-0.225	-0.007		
MGVS	0.263	0.061	0.182	0.232	-0.245	-0.244	0.211	
STSD	0.126	0.051	0.103	0.352	0.109	-0.106	-0.891	0.256

Statistically significant relationship between two variables indicates in bold.

^a^*P* < 0.001

^b^*P* = 0.005

^c^*P* = 0.002

^d^*P* = 0.044

^e^*P* < 0.001

**Table 2:** Independent and dependent variables.

Variables	Value^a^	Range
FCR (°)	6.1 ± 2.0	2 to 10
Body mass index (kg/m^2^)	25.1 ± 2.7	19.8 to 31
Preoperative HKA axis (°)	5.0 ± 3.2	−0.3 to 13.9
K-L grade (3/4)	9/19	3 or 4
NaviLDFA (°)	84.0 ± 2.0	79 to 88
XrayLDFA (°)	85.2 ± 1.6	82.1 to 88.8
LGVS (mm)	8.8 ± 2.5	3.3 to 13.8
MGVS (mm)	8.3 ± 1.2	5.0 to 10.9
STSD (mm)	−0.4 ± 2.5	−4.2 to 4.8

^a^Expressed as mean ± standard deviation unless otherwise indicated.

**Table 3:** Backward multiple regression analysis of factors that affect femoral component rotation (FCR) based on the posterior condylar axis. NaviLDFA as an independent variable.

Dependent variable	Step	Predictors included	Predictors excluded	*P* of included predictors	r^2^	Adj r^2^	F	*P*^a^	β	*P*^b^
FCR	1	BMI, Preop HKA axis, K-L grade, NaviLDFA, MGVS, LGVS, STSD		BMI: 0.888, Preop HKA axis: 0.757, K-L grade: 0.484, **NaviLDFA: 0.001,** MGVS: 0.728, LGVS: 0.744, STSD: 0.188	0.503	0.36	3.535	**0.014**		
	2	Preop HKA axis, K-L grade, NaviLDFA, MGVS, LGVS, STSD	BMI	Preop HKA axis: 0.748, K-L grade: 0.482, **NaviLDFA: 0.001,** MGVS: 0.707, LGVS: 0.736, STSD: 0.180	0.502	0.389	4.436	**0.006**		
	3	K-L grade, NaviLDFA, MGVS, LGVS, STSD	Preop HKA axis	K-L grade: 0.493, **NaviLDFA: <0.001,** MGVS: 0.707, LGVS: 0.735, STSD: 0.183	0.5	0.413	5.742	**0.002**		
	4	K-L grade, NaviLDFA, LGVS, STSD	MGVS	K-L grade: 0.507, **NaviLDFA: <0.001,** LGVS: 0.707, STSD: 0.310	0.497	0.434	7.889	**0.001**		
	5	NaviLDFA, LGVS, STSD	K-L grade	**NaviLDFA: <0.001,** LGVS: 0.757, STSD: 0.381	0.487	0.446	11.867	**<0.001**		
	6	NaviLDFA, STSD	LGVS	**NaviLDFA: <0.001,** STSD: 0.174	0.472	0.43	12.179	**<0.001**		
	7	**NaviLDFA**	STSD		0.447	0.426	21	**<0.001**	-0.668	**<0.001**

^a^Statistical significance of the model

^b^Statistical significance of the predictors included in the final model

**Table 4:** Backward multiple regression analysis of factors that affect femoral component rotation (FCR) based on the posterior condylar axis. XrayLDFA as an independent variable.

Dependent variable	Step	Predictors included	Predictors excluded	*P* of included predictors	r^2^	Adj r^2^	F	*P*^a^	β	*P*^b^
FCR	1	BMI, Preop HKA axis, K-L grade, XrayLDFA, MGVS, LGVS, STSD		BMI: 0.878, Preop HKA axis: 0.493, K-L grade: 0.790, **XrayLDFA: 0.025,** MGVS: 0.526, LGVS: 0.769, STSD: 0.795	0.362	0.179	1.984	0.114		
	2	Preop HKA axis, K-L grade, XrayLDFA, MGVS, LGVS, STSD	BMI	Preop HKA axis: 0.471, K-L grade: 0.753, **XrayLDFA: 0.016,** MGVS: 0.524, LGVS: 0.766, STSD: 0.789	0.361	0.216	2.487	0.063		
	3	Preop HKA axis, K-L grade, XrayLDFA, MGVS, STSD	LGVS	Preop HKA axis: 0.451, K-L grade: 0.804, **XrayLDFA: 0.014,** MGVS: 0.477, STSD: 0.789	0.359	0.247	3.22	**0.031**		
	4	Preop HKA axis, XrayLDFA, MGVS, STSD	K-L grade	Preop HKA axis: 0.451, **XrayLDFA: 0.014,** MGVS: 0.128, STSD: 0.781	0.357	0.277	4.445	**0.013**		
	5	Preop HKA axis, XrayLDFA, STSD	MGVS	Preop HKA axis: 0.435, **XrayLDFA: 0.013,** STSD: 0.772	0.344	0.292	6.556	**0.005**		
	6	Preop HKA axis, XrayLDFA	STSD	Preop HKA axis: 0.389, **XrayLDFA: 0.007**	0.328	0.294	7.109	**0.004**		
	7	**XrayLDFA**	Preop HKA axis		0.324	0.298	12.454	**0.002**	-0.714	**0.002**

^a^Statistical significance of the model

^b^Statistical significance of the predictors included in the final model

Preoperative and postoperative patellar tilt angles were not significantly different (*P* = 0.26). No outliers (>10°) were found in the postoperative patellar tilt angles. The postoperative HKA axis was improved as compared with the preoperative axis (Table 5).

**Table 5:** Comparison of preoperative and postoperative patellar tilt angles and the HKA axis.

	Pre Operation	Post Operation	*P*
Patellar tilt angle (°)			
Mean ± standard deviation (range)	5.3 ± 2.2 (−0.4 to 10.1)	5.9 ± 1.9 (0.5 to 8.8)	0.26
Mean difference ± standard deviation	0.62 ± 2.84		
HKA axis (°)			
Mean ± standard deviation (range)	5.0 ± 3.2 (0.3 to 13.9)	0.9 ± 1.6 (−2.3 to 4.6)	**<0.001**
Mean difference ± standard deviation	−4.10 ± 3.09		

## Discussion

The most important finding of the present study was that both the NaviLDFA and XrayLDFA were associated with the femoral component rotation in valgus knee TKA. Every 1° increment with NaviLDFA induced a 0.668° decrement in FCR and a 1° increment with XrayLDFA induced a 0.714° decrement. In gap-technique TKA, the rotational alignment of the femoral component is adjusted on the basis of the mediolateral flexion gap difference [4]. In the present study, neither lateral soft tissue tightness (LGVS) nor mediolateral soft tissue tension difference (STSD) were significant predictors of FCR. We believe there are two possible explanations for this finding. First, the internal rotation of the posterior condylar axis was the overwhelming determining factor of FCR in valgus knee TKA. Second, the STSD did not perfectly reflect the mediolateral flexion gap difference and was measured on stress radiographs with the knee in extension.

Many studies have demonstrated that the OrthoPilot navigation system can help ensure accurate evaluation of the mechanical axis in the frontal and sagittal planes by kinematic registration of the hip, knee, and ankle centers. The range of intraobserver and interobserver errors by landmark registration was 0.1° to 1.3° [8–10]. However, the variations in the radiographic alignment measurements were as high as 4°, with varying combinations of knee flexion and internal/external rotation [11–13]. A previous study demonstrated that NaviLDFA is more precise and better reflects cartilage status than XrayLDFA. Thus, we expected NaviLDFA to have better correlation with FCR. In this study, value of NaviLDFA (84.0 ± 2.0°) was significantly less than XrayLDFA (85.2 ± 1.6°, *P*<0.001), which could be due to the cartilage degradation in the distal lateral femoral condyle.

The rotational alignment of the femoral component directly affects the patellofemoral joint [14–16]. No outliers (>10°) of the postoperative patellar tilt angle were found, nor any significant difference between the preoperative and postoperative patellar tilt angles. These results suggest that proper rotational alignment of the femoral component was achieved in all the cases.

Berger et al initially defined the posterior condylar angle (PCA) as the angle between the surgical transepicondylar and the posterior condylar axes [17]. In general, the posterior condylar axis is 3° internally rotated relative to the surgical transepicondylar axis [18–20]. The PCA is a relatively consistent measurement in varus deformity with osteoarthritis [18, 21]. However, in valgus deformity, the PCA can vary significantly due to posterior lateral femoral hypoplasia [22–24]. Thienpont et al found that the external rotation of the surgical transepicondylar line relative to the posterior condylar line was higher for femoral valgus than the neutral alignment after analyzing the correlation between the PCA and the mechanical femur axis measured on preoperative computed tomography (CT) scan [25]. Luyckx et al demonstrated a linear relationship between the coronal and rotational geometries of the distal femur on preoperative CT scan. They asserted that every 1° in coronal alignment from varus to valgus induced a 0.1° change in the PCA. While many factors contribute to the need for femoral component rotation adjustment in navigation-assisted TKA, the results of our study suggest that the femoral component rotation mainly reflects the PCA in valgus knee TKA. A similar trend was observed between the FCR and the HKA axis, although this was not statistically significant (*P* = 0.069 from the Pearson correlation analysis). Two previously published studies were not conducted to investigate the correlation between the LDFA and the PCA. Their studies obtained coronal alignment of the lower extremity from CT images performed in the non-standing position. In the present study, the HKA axis correlated with the LDFAs and the LDFAs correlated with the FCR. We agree with the trend of the previous results, but our results demonstrated a stronger correlation between the LDFA and FCR than with the HKA axis.

A lower LDFA indicates greater hypoplastic deformity of the distal lateral condyle, while a higher FCR indicates greater hypoplastic deformity of the posterior lateral condyle. Our results therefore suggest that symmetric lateral condylar hypoplasia occurs in the posterior and distal sides in end-stage osteoarthritis with valgus deformity. To the best of our knowledge, no previous studies have investigated this relationship between distal lateral condylar hypoplasia and posterior condylar hypoplasia.

Some studies have recommended preoperative CT prior to TKA to obtain the individual PCA for each patient [18, 25, 26]. A CT scan, however, cannot show the residual cartilage of the posterior condyle. Some studies have demonstrated that unequal cartilage between the medial and lateral condyles in osteoarthritis can cause an error in determining the PCA [19, 27]. Preoperative CT scan is advantageous for adjusting the femoral component rotational alignment, but we believe it is not essential when valgus knee TKA is performed with a navigation system. Measuring the LDFA before adjusting the femoral component rotation would be beneficial without increasing the radiation exposure or cost.

The present study has some limitations. First, patients who underwent lateral soft tissue release were excluded, so patients with excessive knee valgus or severe lateral tightness were not evaluated. Second, the FCR might have had a measurement error due to our acceptance of a minor mediolateral flexion gap difference in the planning stage (<2 mm). Third, the sample size is not large, so more cases are needed for evaluating a more exact relationship. Despite these limitations, our study is the first to evaluate the correlation between femoral component rotation and predictive factors of valgus knee with end-stage osteoarthritis. This is also the first study to suggest symmetrical lateral condylar hypoplasia in valgus knee with end-stage osteoarthritis.

## Conclusions

Lateral distal femoral angles, as determined using radiograph and the navigation system, were predictive factors of rotational alignment of the femoral component based on the posterior condylar axis in gap-based TKA for valgus knee. Every 1° increment with NaviLDFA leads to a 0.668° decrease in FCR, and a 1° increment of XrayLDFA leads to a 0.714° change. This suggests that symmetrical lateral condylar hypoplasia of the posterior and distal sides occurs in the lateral compartment in end-stage osteoarthritis with valgus deformity.

## Supporting information

S1 File IRB checklist. (PDF)

S2 File Dataset 1. (SAV)

S3 File Dataset 2. (SAV)
