# Supplementary material for: Factors affecting femoral rotational angle based on the posterior condylar axis in gap-based navigation-assisted total knee arthroplasty for valgus knee
Source: PLoS One. 2018 May 15;13(5):e0197335. doi: 10.1371/journal.pone.0197335 (PMC5953479; doi:10.1371/journal.pone.0197335)
Supplement: S1 File — (PDF) [file pone.0197335.s001.pdf]

# NOTIFICATION LETTER

# OF SMC IRB REVIEW SUMMARY

| | |
|---|---|
| Investigator : | Moon, Young-Wan. M.D., Ph.D.<br>Professor, Dept. of Orthopedic Surgery, SMC |
| SMC IRB File No. : | 2017-05-049-003 |
| Protocol Title : | Predictable factors of the femoral rotational angle based on the posterior condylar axis in navigation-assisted total knee arthroplasty for valgus knee |

☑ **Approval** ☐ Contingent approval

* Please append details or summarize reason(s) for determination below:
SMC IRB concluded that your alternation plan meets our previous recommendations. In addition, SMC IRB confirmed that protocol amendments, which has been reviewed by expedited review process and it seems not to involve increased risks to the research participants.

It is determination of SMC IRB that the continuing review period shall be 12 months considering risk and benefit ratio for the research participants. Acquiring participant's consent (1) seems to be realistically impossible and does not influence integrity of research (2) there would be no reasons that participant would deny providing his or her consent, even though consent is waivered, research involves no more than minimal risk to the participants. Therefore, participant's consent can be waivered.

The final IRB determination for this research is "Approval" for one year.
To continue the research after the expiration of IRB approval, the PI must submit "Research Progress Report (www.e-IRB.com → Log-in → e-IRB Main Bar → Download Forms → Reference Room)" for the continuing review after Apr. 01, 2018.

**Date of Review : Jun. 02, 2017**

Institutional Review Board
Samsung Medical Center

#81, Irwon-Ro, Gangnam-Gu, Seoul, Korea, 135-710

# CONTENTS OF IRB REVIEW

| | |
|---|---|
| Investigator : | Moon, Young-Wan. M.D., Ph.D.<br>Professor, Dept. of Orthopedic Surgery, SMC |
| SMC IRB File No. : | 2017-05-049-003 |
| Protocol Title : | Predictable factors of the femoral rotational angle based on the posterior condylar axis in navigation-assisted total knee arthroplasty for valgus knee |

**DOCUMENT REVIEWED**

- Research Protocol

Issued by

Rhee, Younjin, IRB Manager
IRB Secretary for Administration
Samsung Medical Center

Feb. 13, 2018
Date

## IRB Information

| | |
|---|---|
| Institution : | Samsung Medical Center (SMC) |
| Address : | #81 Irwon-ro, Gangnam-gu, Seoul, Korea |
| IRB President : | Prof. Suk-koo Lee, M.D., Ph.D.<br>Dept. of Pediatric Surgery |

SMC IRB abides by the guideline of ICH and GCP.

SMC IRB and the Institution are registered at Office for Human Research Protections (OHRP), U.S. Dept. of Health & Human Services, U.S.A..

Related SMC IRB registration and accreditation information is as follows.

* Identifier No. (U.S. OHRP)

| | |
|---|---|
| Organization : | IORG0000545 - Samsung Med Ctr |
| Assurance : | FWA00002750 |
| IRBs : | IRB #1 : IRB00000877<br>IRB #2 : IRB00005489<br>IRB #3 : IRB00005490<br>IRB #4 : IRB00005491<br>IRB #5 : IRB00006642<br>IRB #6 : IRB00006643<br>IRB #7 : IRB00008480<br>IRB #8 : IRB00008481<br>IRB #9 : IRB00011389<br>IRB#10 : IRB00011390 |

* Samsung Medical Center has earned the accreditation from the Association for the Accreditation of Human Research Protection Programs, Inc. (AAHRPP) on June 16, 2006.

This research protocol is reviewed by expedited IRB review process
